# Validity and time course of surgical fear as measured with the Surgical Fear Questionnaire in patients undergoing cataract surgery

**DOI:** 10.1371/journal.pone.0201511

**Published:** 2018-08-09

**Authors:** Maurice Theunissen, Soraya Jonker, Jan Schepers, Nancy A. Nicolson, Rudy Nuijts, Hans-Fritz Gramke, Marco A. E. Marcus, Madelon L. Peters

**Affiliations:** 1 Department of Anesthesiology and Pain Management, Maastricht University Medical Center+, Maastricht, the Netherlands; 2 Department of Ophthalmology, Maastricht University Medical Center+, Maastricht, the Netherlands; 3 Department of Methodology & Statistics, Maastricht University, Maastricht, the Netherlands; 4 Department of Psychiatry & Neuropsychology, Maastricht University Medical Center+, Maastricht, the Netherlands; 5 Department of Anesthesia/ICU, Pain & Palliative Care, Hamad Medical Corporation, Doha, Qatar; 6 Department of Clinical Psychological Science, Maastricht University, Maastricht, the Netherlands; Public Library of Science, UNITED KINGDOM

## Abstract

**Objectives:**

The primary aim of the study was to assess the convergent validity of the Surgical Fear Questionnaire (SFQ) with other self-report instruments and biological indices of stress. Secondary aims were the examination of predictors of the level and time course of fear and preferences for fear treatment.

**Methods:**

In a prospective observational cohort study SFQ short-term (SFQ-s) and long-term (SFQ-l) scores were assessed one week, one day, and the morning before cataract surgery, together with salivary cortisol and alpha-amylase (sAA) levels, and numeric rating scale (NRS) fear score. SFQ-scores were also assessed before second eye surgery. Expected pain and recovery, and sociodemographic and medico-psychological predictors of fear were assessed at baseline.

**Results:**

Data of 98 patients were analyzed. Scores of both SFQ-subscales (range 0–40) were generally low, all mean ≤ 9.0. SFQ-s and SFQ-l correlated significantly with the other self-report instruments: NRS fear .83 and .89, expected pain .49 and .54, expected recovery -.27 and -.44. No association was found between SFQ-scores and cortisol or sAA level. Predictors of the level of fear were baseline pain and stress. Additional effects of time were found for subgroups based on educational level, antidepressant use, and presurgical stress (SFQ-l). SFQ-scores were significantly lower before the second cataract surgery than before the first, and higher in patients who would have appreciated treatment of fear.

**Discussion:**

Convergent validity of the SFQ with other self-report measures is shown. The sensitivity of the SFQ permits the detection of small variations in fear caused by time or other factors.

## Introduction

Across several studies surgical fear has been associated with increased postoperative pain, increased use of analgesia, and poor postoperative recovery, physical functioning, or mental health [1–6]. As a result, assessment of surgical fear seems important because it is the first step towards preoperative treatment of fearful patients which, in turn, is likely to increase postoperative personal well-being and might save considerable medical and societal costs.

For a valid assessment of surgical fear two aspects need consideration: the selection of an adequate instrument and the timing of assessment. Previous studies have used a variety of instruments for the assessment of surgical fear: general instruments such as the State Trait Anxiety Inventory (STAI) [7] and Hospital Anxiety and Depression Scale (HADS) [8] or disease specific instruments such as the Bypass Graft Fear Scale (BGFS) [9,10]. This publication will focus on another questionnaire, dedicated to asses surgical fear across all types of clinical and day surgery patients, the Surgical Fear Questionnaire (SFQ) [11]. The SFQ is formatted as an eight item index of surgical fear which can be divided into fear of short- and long-term consequences of surgery. Initial validation of the SFQ showed it to be a valid and reliable questionnaire, however, so far all studies administered the questionnaire at just one time point. As a result, information about the optimal time point for assessing surgical fear, the sensitivity of the SFQ to detect changes in fear over time, and factors that may influence the course of fear, is lacking. Therefore, in the present study we administered the SFQ at multiple time points before cataract surgery, i.e. one week before the scheduled procedure, the night before and at the morning of surgery.

Boker et al. (2002) found a significant increase in surgical fear in patients undergoing various types of surgery after admittance to the surgical ward, compared to assessment during their visit to the pre-anesthesia clinic [12]. However, a previous study in cataract patients did not find a difference in state anxiety one to two weeks before surgery compared to just before surgery [13]. In the latter study, a generic state anxiety instrument was used, and the SFQ may prove more sensitive to detect subtle changes in surgery-related fear specifically. Moreover, since most cataract patients are scheduled for bilateral cataract surgery, this enables comparison between surgical fear of the first versus the second cataract surgery. Usually recovery is unproblematic and most patients feel relieved after surgery [13]. We expected an increase in fear from one week before surgery to the assessment the night and the morning before surgery. In addition we expected that fear before the second surgery would be lower than before the first surgery. Finally, previous data have shown that preoperative pain is associated with increased surgical fear [14,15]. Therefore a study population with a known low prevalence of preoperative pain is preferred to minimize confounding effects on SFQ scores. Cataract surgery is known for having a low prevalence of preoperative pain [15].

Besides information on the validity and sensitivity of the SFQ with regard to effects of time course, we assessed convergent validity of the SFQ with other self-report instruments (numerical rating scale of fear, expected pain, and expected recovery) and with biological indices of stress (cortisol, alpha-amylase). Cortisol is used as a biomarker of activity of the hypothalamic-pituitary-adrenal (HPA) axis [16]. Cortisol secretion shows a circadian rhythm with high morning levels and declining levels during the day; its secretion can be increased by physical or mental stress. We hypothesized that SFQ scores would predict cortisol elevation on the day of surgery compared to cortisol level on a control day. Cortisol can be measured in saliva where it serves as a good indicator of the serum unbound and active cortisol fraction. Salivary alpha-amylase (sAA) can be used as an indicator of activation of the autonomic nervous system. Previous studies showed good correlation between sAA levels and acute stress [17,18]. We hypothesized that SFQ scores would predict a greater increase in pre- to postoperative sAA levels.

As a secondary aim, we assessed predictors of the level and time course of fear. Based on the findings of previous studies, we expected fear to be higher in women, in patients with high levels of preoperative pain, in patients with generally high psychological vulnerability, and in patients reporting low social support [11,13,19–21]. Age, employment status, and educational level were included as additional factors that might be related to fear level and course, since these have been found predictive of surgical fear in some studies [11].

In sum, we assessed sensitivity of the SFQ to detect differences in the preoperative time course of fear and sensitivity to detect changes in fear from first to second surgery. We hypothesized fear to increase closer to the time of surgery, and to be lower before the second surgery. Convergent validity was assessed by establishing the association of the SFQ with related self-report measures and with biological indices of stress. Finally we examined potential predictors of the level and time course of surgical fear.

## Materials and methods

### Ethics statement

Prior to the conduction of this study, approval was obtained from the Medical Ethics Committee of the Maastricht UMC+, Maastricht, the Netherlands. Signed informed consent was obtained from all participants and the study was conducted according to the declaration of Helsinki. This study was registered at the Dutch Trial Register under number NTR4491.

### Patient recruitment and sample size

Patient recruitment took place at the University Eye Clinic at the Maastricht UMC+, Maastricht, the Netherlands. Inclusion criteria were age over 18 years, scheduled for elective cataract surgery in a day surgery setting, (loco-)regional anesthesia, a good command of the Dutch language, an ASA classification varying from I to III, and time to surgery of over 7 days. Exclusion criteria consisted of previous cataract surgery on the contralateral eye during the past year, illiteracy, cognitive impairment, surgery scheduled under general anesthesia, ASA classification IV, Morbus Cushing, Addison’s disease, hypothyroidism, hyperthyroidism, corticosteroid use (except inhaler), and participation in another trial.

If the true difference between mean SFQ scores assessed one week before surgery and assessed on the morning on the day of surgery is five, we would need to study 93 subjects in order to be able to reject the null hypothesis that this response difference is zero with a power set at 0.8. Based on prior data [11,15] we assumed a standard deviation of 17 for the difference between SFQ scores. The Type I error probability associated with this test of this null hypothesis was 0.05. With an additional nine patients for correction of drop-out, 102 patients would need to be included.

### Measurements

#### Surgical Fear Questionnaire (SFQ)

The SFQ is an eight-item instrument for the assessment of self-reported surgical fear, suitable for general use among all types of adult surgery patients, covering a range of short-term (SFQ-s, item 1–4) and long-term (SFQ-l, item 5–8) surgery-related fears. All items are scored on an eleven point numeric rating scale (NRS) ranging from 0 (not at all afraid) to 10 (very afraid). This results in a score of 0–40 for each subscale and a total score of 0 to 80. Items are: afraid of operation, anesthesia, postoperative pain, side effects, health deterioration, failed operation, incomplete recovery, and long duration of rehabilitation. See S1 File.

### Measures for assessing convergent validity

#### Fear NRS

Pre- and post-operative fear was assessed with one question: “Circle the number that best reflects how afraid you are right now”, that was scored on a 11-point scale (0–10). Labels were not at all afraid (0) and very afraid (10).

#### Expected pain

The level of pain patients expected to have after surgery was assessed with the question “Please indicate on a scale from 0 (no pain at all) to 10 (worst pain imaginable) how much pain you expect to feel four days after surgery”. It was also scored on a 11-point scale and assessed at baseline, one week before surgery [22]. Expected pain at day four was chosen because, in general, acute pain levels at day four are expected to be low [23]. If pain levels at day four are high, this may be indicative of poor recovery.

#### Expected recovery

Expected recovery after surgery was also assessed at baseline, using the question “Please indicate on a scale from 0 to 100% to what extent you expect to be recovered from the operation, four weeks after the operation. 100% recovery means that your health is back at the level it was before you developed your complaints and surgery was performed”. This question was adapted from the Global Surgical Recovery scale (GSR) [24]. Another measure of expected recovery was the anticipated number of days until resumption of daily activities. Patients were asked at baseline to enter a number of days in answer to the question: “After how many days do you expect to be able to completely resume your daily activities?”.

#### Cortisol and alpha-amylase

Saliva samples were obtained using Salivette swabs (Sarstedt AG & Co., Nümbrecht, Germany). The synthetic swabs were specifically designed for cortisol determination in a small amount of saliva. Soft chewing on the swab during 60 seconds stimulates salivary production and ensures absorption of a sufficient amount of saliva. For the saliva samples collected at home, patients were instructed to collect the morning samples immediately after awaking, before brushing teeth or having breakfast, and for the other samples to refrain from smoking, eating and drinking for a minimum of 30 minutes, and in case of alcohol intake a minimum of two hours, prior to sample collection. Patients were asked to store the swabs in their home freezer before handing them in at the hospital visits. In the hospital saliva samples were frozen and stored in a freezer at -20° C until shipment to and analysis at the laboratory of the Technical University of Dresden, Germany. After thawing, Salivettes were centrifuged at 3,000 rpm for 5 minutes, resulting in a clear supernatant of low viscosity. Salivary concentrations were measured using commercially available chemiluminescence immunoassay with high sensitivity (IBL International, Hamburg, Germany). The intra- and interassay coefficients of variation for cortisol were below 8%.

The Salivettes additionally allowed for salivary alpha-amylase (sAA) assessment, performed in the same lab in Dresden. Concentration of alpha-amylase in saliva was measured by an enzyme kinetic method, as described by Rohleder et al. (2006) [25].

### Measures for examining predictors of surgical fear

#### Sociodemographic data

A baseline questionnaire completed one week before surgery assessed age, sex, marital state (living alone versus living together/married), employment state (full-time or part-time work, temporary unable to carry out paid job, retired, student, unemployed, disabled, housekeeping, other), educational level (no, lower, intermediate, higher, university), smoking (yes/no/stopped), alcohol use (yes/no), and medication use. The following groups of medications were considered for the analyses of surgical fear and cortisol: total number of home medication, COX-2 inhibitors, antidepressants, anxiolytics, hypnotic sedatives, nonsteroidal anti-inflammatory drugs, inhaled steroids, antihypertensives, anticonvulsants [26].

#### Pre-surgical pain

Pain present prior to surgery was assessed separately for pain related and unrelated to the cataract surgery. At first patients were asked whether they had pain last week or not. If so, a pain intensity score reflecting any pain (related or unrelated to the eye) at the moment of completing the questionnaire was assessed with a 0–10 numerical rating scale [22]. The following baseline pain data were also assessed but not further analyzed: average and highest pain intensity during the last week, the onset of pain, the location of pain, and in addition to analgesic use, non-pharmaceutical pain treatment.

### Social support

#### Social support was measured with the medical outcomes

Study Social Support Survey (MOS-SSS) questionnaire [27]. The MOS-SSS is a 19-item self-report questionnaire with four support subscales: emotional/informational, tangible, positive interaction, and affectionate support. In addition an overall support index can be calculated. One extra question assesses the number of close friends or relatives. Items are scored on a five-point scale. For the present study we used the total score, which is scaled from 0–100. Social support served as one of the control factors for stress, based on the assumption that patients with more social support might experience lower stress levels.

#### Stress

Stress was assessed at baseline using the Perceived Stress Scale (PSS) [28]. The PSS is a valid and reliable 10-item self-report measure of the degree to which life situations are appraised as stressful over the past month. Items are scored on a five-point scale. After rescaling of four positively worded items, a total score from 0–40 can be obtained by summation of the item scores. In addition, on the day of surgery patients were asked whether they had experienced a major event that caused stress in the week before surgery. Answer categories were no; yes, a little bit stress; yes, much stress; yes, very much stress.

### Other per- and postoperative measures

#### Day of surgery

Body mass index (BMI), American Society of Anaesthesiologists (ASA) classification, co-morbidity, type of surgical intervention, type of local analgesia, duration of operation, perioperative complications, and unplanned hospital admission were assessed. Only data on ASA and hospital admission are reported.

#### Post-surgery questionnaire

At the final visit four weeks post-surgery patients handed in a final questionnaire completed one day earlier, assessing recovery as measured with the GSR. Patients responded to the question how much they felt recovered compared to their situation before surgery (0–100%). Patients were also asked if they were able to perform their daily activities again since the last surgery, and if true, the number of days until that moment. Finally, postoperative pain intensity was assessed using the NRS.

Furthermore we retrospectively assessed whether patients would like to have had additional therapy to treat their surgical fear. The question was: “Thinking back to the period from one week before surgery until the day of surgery: would you have appreciated any treatment of surgical fear?” Possible answers were: “No, I was not (very) afraid and I don’t appreciate treatment”, No, I was afraid, however I don’t need treatment”, or “Yes, I was afraid and would have appreciated treatment”. Patients who answered “yes” were able to choose one or more of the following treatment categories they would have preferred: more information provided by the ophthalmologist or the anesthesiologist, counselling by a psychologist, relaxation exercises, guided visualization, meditation, pharmaceutical treatment, or other.

To control for events that might affect the cortisol levels, the occurrence of major stressful events other than the operation during the four postoperative weeks was assessed. Participants were asked: “during the past four weeks (after surgery), did you go through anything that caused a lot of stress?” Response categories were “no”; “yes, but just a little bit of stress”; “yes, much stress”; “yes, very much stress”.

### Procedure

**Fig 1:**
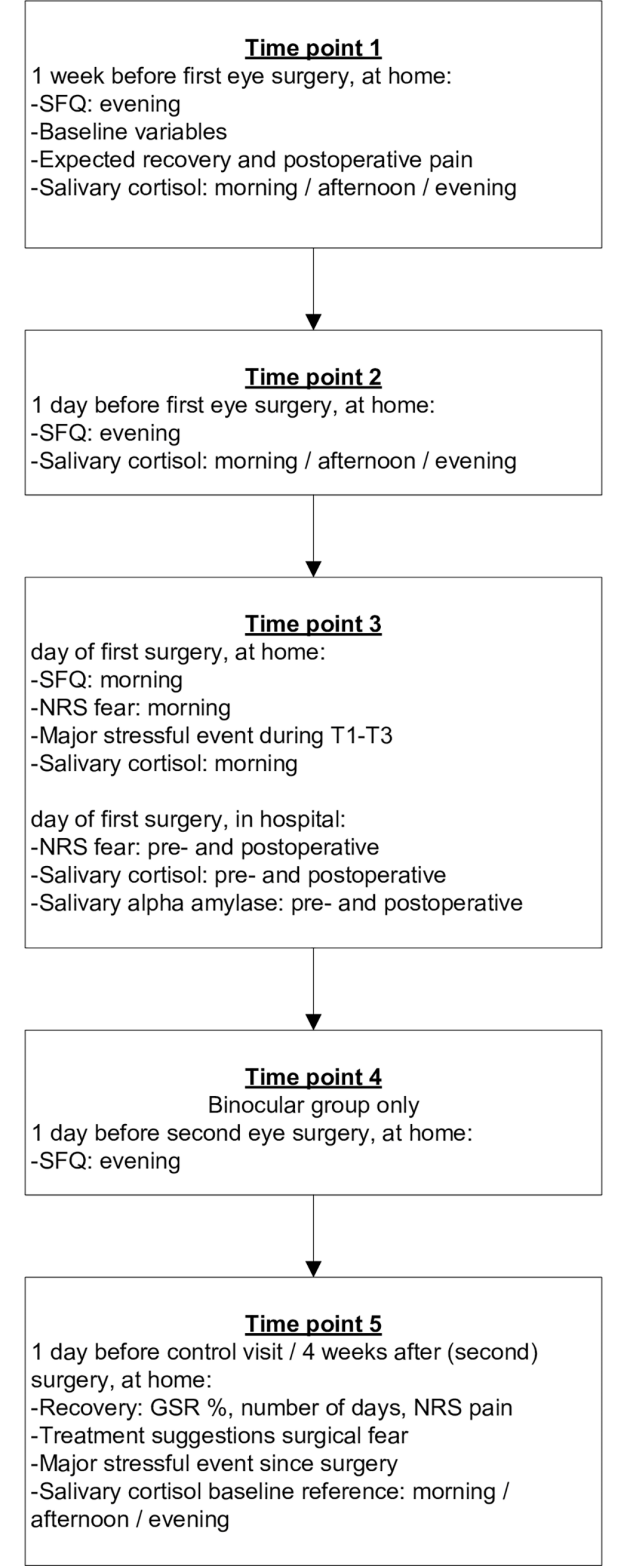
Schedule study measurements.

Patients received a batch of date- and time-marked questionnaires and Salivette swabs the day they signed their informed consent form and the day of their first surgery. They were instructed to fill out the questionnaires and use the swabs at the indicated days and times, and for control, to register the exact dates and times of completion and saliva collection. The first SFQ was filled out on the evening one week before surgery (T1). At this time, patients also completed the baseline questionnaire. The second SFQ assessment took place on the evening before surgery (T2). On both of these days, patients were asked to collect three saliva samples, i.e., directly after awakening, in the afternoon between three and four o’clock, and in the evening between eight and nine. The third SFQ was filled out at home after awaking, on the morning surgery took place (T3). At this time, patients also scored fear on the NRS and took another saliva sample. In the hospital, two more NRS fear ratings were made, and at the same time saliva samples were taken: before surgery immediately after arrival at the ward of the University Eye Clinic, and after surgery when the patient had returned to the ward. Patients scheduled for cataract surgery on both eyes filled out the SFQ a fourth time, i.e., the evening before their second surgery (T4). Finally all patients returned to the hospital four weeks after (second) surgery for a control visit. The day before this control visit patients filled out the post-surgery questionnaire, containing questions on recovery, pain, and need for treatment of surgical fear (T5). Also, saliva samples were taken on that day, to serve as a baseline reference for cortisol. For an overview of the procedure see Fig 1.

Cortisol was analyzed from the three saliva samples obtained on the two preoperative days, the morning sample and pre- and post-surgical samples on the day of the (first) surgery and from the three samples of the control day four weeks following surgery. Because sAA is thought to increase mainly in response to acute stressors, sAA was only analyzed in the immediate pre- and post-surgical samples on the day of surgery.

Data obtained by questionnaires were entered into a database in duplo by independent members of the study staff. Both databases were compared and all inconsistencies were corrected.

### Statistical analysis

Descriptive analysis was performed on baseline and outcome data, using mean ± sd, median (25^TH^-75^TH^ percentile), number (%), or boxplots. To evaluate SFQ-s and SFQ-l scores over time bivariate analyses were performed using paired t-tests. We assessed whether there was an increase between T1 (one week before surgery) and T3 (the morning on the day of surgery), and for the binocular group we assessed whether there was a decrease between first surgery (T2, the evening before first surgery) and second surgery (T4, the evening before second surgery).

Convergent validity of the SFQ was assessed by calculating Pearson’s correlation coefficients between SFQ-s and SFQ-l at T3 and the preoperative NRS fear scores, also completed at home at T3. Furthermore correlations were assessed between SFQ-s and SFQ-l at T1, and expected pain, expected recovery (GSR), and expected number of days until resumption of daily activities. To assess convergent validity of the SFQ with the biomarkers cortisol and sAA, multivariate analyses of natural-log transformed cortisol and sAA levels were performed. For cortisol, the differences between the morning samples of the day of surgery and the control day 4 weeks later (T3-T5) were regressed on SFQ-s and SFQ-l in separate analyses. For sAA, the differences between the immediate pre- and post-surgical levels were calculated and regressed on SFQ scores. Potential confounding factors such as sociodemographic variables, smoking habits, health status (defined by ASA-classification), medication use (total number of home medications, hypnotic sedatives, inhaled steroids), and the time at which the sample was taken were added to the model [29,30]. Results of the regression analyses are presented as beta (sd).

To examine predictors of the overall level and the time course of surgical fear before the first surgery, multivariate analysis was performed using linear mixed modeling with a random person intercept and time variables defining T2 and T3, while T1 served as a reference. Age, sex, employment status (recoded to paid job yes/no), educational level (recoded to no/lower, intermediate, or higher/university education), pain at baseline (NRS ≥ 4 versus < 4), social support (total score), perceived stress during the month prior to T1 as measured with the PSS, and stress during the week between T1-T3 (no/little stress versus (very) much stress) were included as predictor variables. We also included the use of anxiolytics and antidepressants as reported on the medical file as additional indicators of psychological vulnerability. Interaction effects between time and each of the selected variables were assessed per variable, for SFQ-s and SFQ-l separately. In the case of a significant interaction effect, the interaction terms were added to the multivariate model (criterion *p* < .10). The time variables, age, and sex were entered using a forced entry method, for all other variables a backward deletion procedure was used (criterion *p* < .10). A final selection criterion of *p* < .05 was used. A significant interaction with time reflects an effect of the respective variable on time course, while a significant main effect implies an overall effect of the respective variable on surgical fear, irrespective of time. Results are presented as estimate (sd), which reflect the change in outcome for each unit of change in the predictor.

A post-hoc analysis was performed to assess whether the SFQ-s and SFQ-l were related to patients’ need for preoperative treatment of fear. Using independent t-tests, the SFQ-s and SFQ-l scores of patients indicating a need for additional psychological treatment for surgical fear were compared to the scores of patients not reporting this need. For all analyses a final *p*-value < .05 was considered statistically significant. Analyses were performed using IBM SPSS Statistics for Windows, version 23.0. Armonk, NY, USA.

## Results

**Fig 2:**
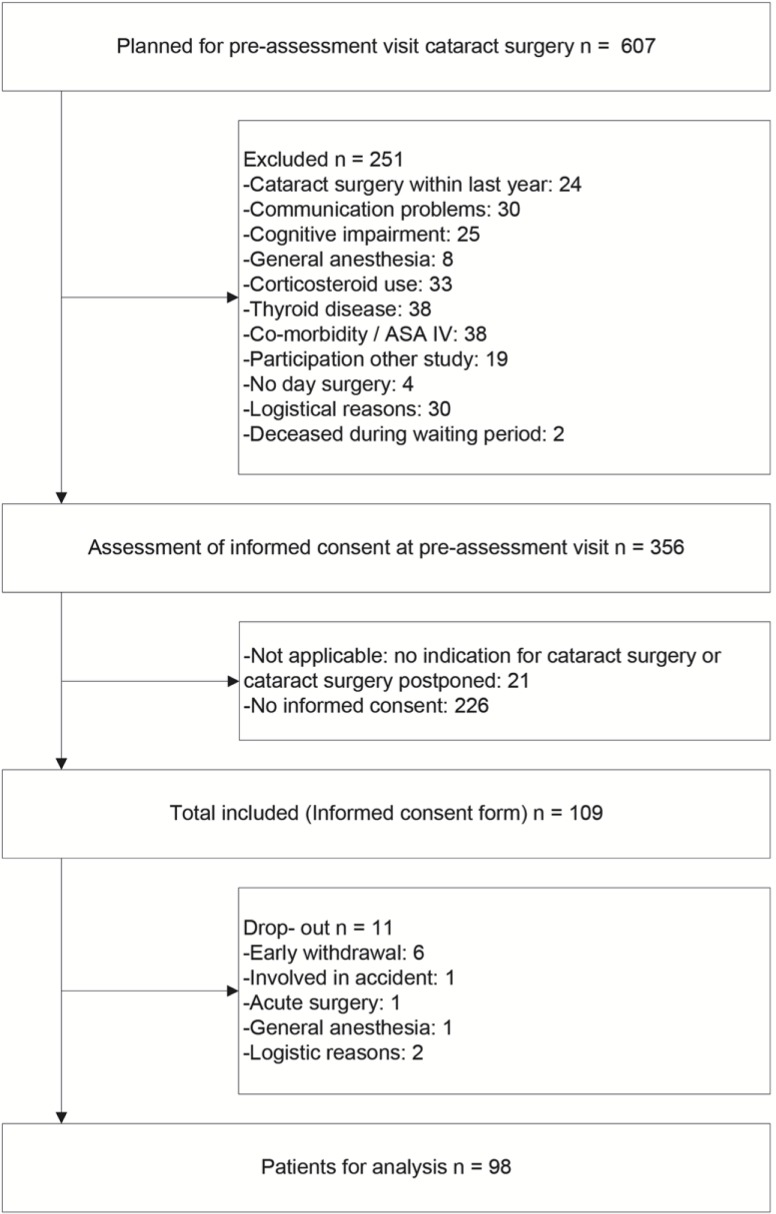
Patient flow.

Between March 2014 and January 2015 109 cataract surgery patients were included, of whom 98 (89.9%) were suitable for analyses. Detailed information on patient flow is presented in Fig 2. Mean (sd) age of the participants was 68.8 (8.9) years, 42 (42.9%) were female, and 49 (50.5%) were scheduled for cataract surgery on both eyes. Patients who underwent cataract surgery on the contralateral eye during the last year were excluded, however there were 17 (17.7%) patients who reported other eye surgery during the last year. Detailed information on the baseline characteristics is presented in Table 1.

**Table 1:** Baseline characteristics.

Variable	N = 98
Age	
	68.8 (8.9)
Sex	
male	56 (57%)
female	42 (43%)
Education	
no / lower	17 (18%)
intermediate	36 (38%)
higher	42 (44%)
missing	3
Employment	
paid job	20 (21%)
no paid job	76 (79%)
missing	2
Marital status	
living alone	27 (28%)
living together	69 (72%)
missing	2
Expected pain (0–10)	2.0 (0–3)
Expected GSR (0–100%)	91.9 (14.4)
Expected number of days	14.0 (14.7)
MOS-SSS total (0–100)	78.2 (23.5)
PSS (0–40)	10.9 (6.7)
Non-surgical stress	
no	52 (56%)
a bit	27 (29%)
a lot	12 (13%)
extreme	2 (2%)
missing	5
ASA	
I	20 (21%)
II	65 (66%)
III	13 (14%)
Smoking	
no	31 (32%)
stopped	59 (62%)
yes	6 (6%)
missing	2
Alcohol	
yes	76 (79%)
no	20 (21%)
missing	2
Medication	
anxiolytics	5 (5%)
antidepressants	5 (5%)
hypnotic sedatives	6 (6%)
inhaled corticosteroids	7 (7%)
antihypertensives	44 (45%)
Previous eye surgery last year	17 (18%) [2]
Planned cataract surgery	
monocular	48 (49%)
binocular	49 (51%)
missing	1
Preoperative pain	
related to eye	2 (2%) [1]
unrelated to eye	36 (37%) [1]

Mean (sd), median (25^th^– 75^th^ percentile), number (%) [missing data].

Expected pain: expected pain four days after surgery (numeric rating scale, NRS 0–10); Expected GSR: expected global surgical recovery at four weeks after last surgery (T5); Expected number of days: expected number of days until full recovery after last surgery. MOS-SSS number: medical outcome study social support scale, number of close friends/relatives; MOS-SSS total: total score (0–100). PSS: perceived stress scale; Non-surgical stress: stress unrelated to surgery with major impact during the week before surgery (the week of T1-T3). ASA: American Society of Anesthesiologists. Preoperative pain: pain last week, yes/no.

Postoperative recovery was generally rated as good. Four weeks after cataract surgery perceived recovery on the GSR was 89.9% (sd 17.7), average pain in the eye during the last week was 0 (median, 25-75^th^ percentile 0–1), 83 patients (89.2%) were able to perform their daily activities again (missing data 5), and the number of days before they were able to perform daily activities again since the last surgery was 6.9 (7.1). There were no unplanned hospital admissions.

### Time course of surgical fear

**Fig 3:**
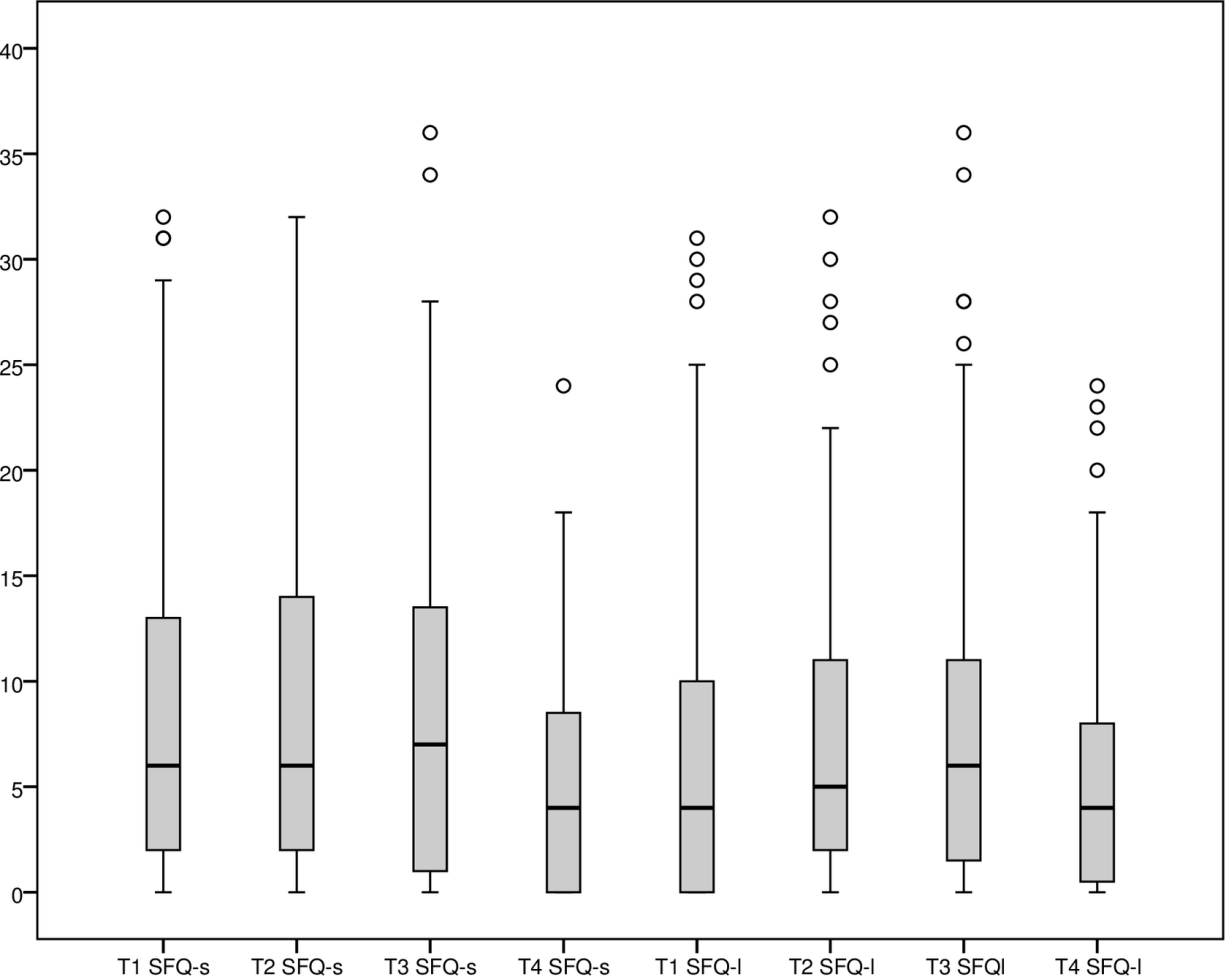
Surgical fear short- and long-term subscales, at time point 1–4. T1-3: all patients, T4: binocular patients only. T1: one week before surgery, T2: the evening before surgery, T3: the morning of the day of surgery, morning, at home, T4: binocular patients, the evening before their second surgery. ° indicates outlier.

Our data revealed low surgical fear scores at all time points for most patients. A minority of patients did however report considerable levels of fear, with a maximum of 36 for both SFQ-s and SFQ-l at T3. Mean (sd) SFQ-s scores were 8.2 (8.4) at T1 and 8.6 (8.7) at T3. The SFQ-l scores were 6.9 (8.1) at T1 and 8.1 (8.5) at T3. A paired t-test revealed that the increase between T1 and T3 was significant for SFQ-l (*p =* .034) but not for SFQ-s. At T2, the evening before the first surgery, the SFQ-s score of the total sample was 8.7 (8.5), SFQ-l was 7.7 (7.7). For the binocular group SFQ-s at T2 was 9.0 (sd 8.6) and decreased to 5.3 (5.8, *p =* .002) at T4, the evening before the second surgery. SFQ-l decreased from 8.0 (7.7) to 5.6 (6.2, *p =* .010). Details on SFQ results are presented in the boxplots of Fig 3.

### Convergent validity of the SFQ with NRS fear scores and preoperative expectations

On the day of surgery the NRS fear score at home was 2.6 (2.6) and pre- and postoperative in-hospital scores were 2.7 (2.4) and 0.8 (1.4) respectively. Convergent validity of the SFQ was assessed using the preoperative NRS fear score assessed at home, and outcome expectancy scores. The Pearson correlations between SFQ-s and SFQ-l scores at T3 and preoperative NRS fear were high, .898 and .828, and significant at alpha = .01 level. The correlations of SFQ scores of T1 with expected pain were somewhat lower, .543 for SFQ-s and .493 for SFQ-l, but also significant at alpha = .01 level. Finally, negative correlations with expected recovery were found, -.267 significant at alpha = .05 level for SFQ-s and -.439 for SFQ-l, significant at alpha = .01 level. The expected number of days until reuptake of activities did not correlate with any of the SFQ scores; no data shown.

### Convergent validity of the SFQ with salivary cortisol and alpha-amylase

The cortisol levels sampled at T1-3 and T5 showed normal circadian patterns with levels decreasing by approximately 2 nmol/L per hour from morning through evening measures. Mean collection times complied with the given patient instructions, see S1 Fig and S1 Table. On average, the samples were collected one hour (sd 1.3) earlier on the day of surgery compared to the day before the control visit. Linear regression analyses were performed using the calculated difference between the natural-log transformed cortisol sample collected at home on the day of surgery and the morning cortisol sample of the day before the control visit as dependent variable, and SFQ scores as independent variables. Furthermore we corrected for potential confounders by including age, smoking, medication use (hypnotic sedatives, inhaled corticosteroids), ASA-classification, and both cortisol sampling times in the analyses. Analyses were performed separately for SFQ-s and SFQ-l. Change in cortisol level was predicted by time of day of the saliva collected at the day of surgery (T3 morning sample). Neither SFQ-s or SFQ-l, nor any of the mentioned confounders contributed significantly to the model, see Table 2. Additional correction for the potential confounders stress and social support did not change these findings. Also the association between the SFQ and the cortisol samples collected at other time points was explored, however no association was found.

**Table 2:** Multivariate assessment of the association between surgical fear and salivary cortisol / alpha-amylase.

		Cortisol		Cortisol		Alpha Amylase		Alpha Amylase	
Predictor		SFQ-s		SFQ-l		SFQ-s		SFQ-l	
		Beta (sd)	*p*	Beta (sd)	*p*	Beta (sd)	*p*	Beta (sd)	*p*
SFQ-s T3		-.002 (.008)	.768	NA		.006 (.009)	.511	NA	
SFQ-l T3		NA		-.010 (.008)	.246	NA		.004 (.008)	.654
Age		.007 (.009)	.435	.003 (.008)	.682	-.004 (.009)	.635	-.006 (.009)	.484
Smoking	no	Reference		Reference		Reference		Reference	
	yes	-.633 (.433)	.149	-.502 (.422)	.238	.148 (.413)	.722	.121 (.405)	.766
	stopped	-.068 (149)	.648	-.036 (.143)	.804	-.037 (.163)	.821	-.025 (.157)	.873
Alcohol use		NA		NA		-.156 (.200)	.441	-.150 (.194)	.444
Marital status		NA		NA		-.215 (.170)	.210	-.212 (.155)	.174
Inhaled steroid use		-.381 (.408)	.354	-.516 (.388)	.188	.108 (.361)	.766	.117 (.353)	.742
Hypnotic sedative use		-.450 (.480)	.352	-.283 (.365)	.441	-.046 (.563)	.935	.178 (.415)	.670
Antihypertensive use		NA		NA		-.040 (.166)	.812	-.043 (.153)	.782
Antidepressant use		NA		NA		.134 (.339)	.694	.136 (.320)	.672
Anxiolytic use		NA		NA		-.542 (.373)	.151	-.598 (.338)	.082
ASA	I	Reference		Reference		Reference		Reference	
	II	.067 (.189)	.723	.098 (.182)	.592	.116 (.200)	.563	.125 (.195)	.524
	III	-.435 (.258)	.096	-.374 (.252)	.142	.034 (.282)	.906	.060 (.278)	.830
Time saliva T3 M		4.273E-5 (.000)	**.042**	4.130E-5 (.000)	**.045**	NA		NA	
Time saliva T5 M		1.834E-5 (.000)	.273	2.039E-5 (.000)	.210	NA		NA	
Time saliva T3 Pre		NA		NA		-4.001E-5 (.000)	.144	-3.496E-5 (.000)	.187
Time saliva T3 Post		NA		NA		3.834E-5 (.000)	.079	3.129E-5 (.000)	.128
Intercept		-1.897 (.008)	**.028**	-1.690 (.812)	**.041**	.473 (.730)	.520	.717 (.667)	.286

Cortisol: the difference between the natural-log transformed morning samples of the day of surgery and the control day 4 weeks later (T3 M minus T5 M). Salivary alpha amylase (sAA): the differences between the natural-log transformed pre- and post-surgical levels (T3 Pre minus T3 Post). Time: day time of sample collection. ASA: American Society of Anesthesiologists.

The preoperative sAA level at T3 was 196.5 (158.3) U/ml mean (sd) and increased to 258.7 (197.1) postoperatively. A paired t-test on the natural-log transformed data revealed that this increase was significant, t -4.628 df 85 *p* < .001. Linear regression analysis was performed with the calculated difference between the natural-log transformed pre- and postoperative sAA sample as dependent variable. Also for sAA, assessed at the day of surgery, separate analyses were performed for SFQ-s and SFQ-l. We controlled for age, marital status, smoking, alcohol use, medication use (antihypertensives, inhaled corticosteroids, hypnotic sedatives, antidepressants, anxiolytics), health status (ASA-classification), and both sAA sampling times. We found no association between either surgical fear or any of the other co-variables with perioperative change in sAA level, see Table 2.

### Prediction of the level and course of fear

The following variables showed an interaction with time in their association with at least one of the subscales of the SFQ in bivariate analyses, and were therefore added to the multivariate model: educational level, perceived stress during the month before T1, stress during the week between T1-T3, use of antidepressants, and use of anxiolytics. The final results after a backward deletion procedure, for SFQ-s and SFQ-l are presented in Table 3. For SFQ-s significant main effects were found for baseline pain [6.0 (2.2), *p =* .007] and perceived stress during the month before surgery [0.5 (0.1), *p <* .001]. Patients reporting higher pre-surgical pain and stress levels reported overall higher levels of surgical fear. Furthermore the effect of time (T3 versus T1) on surgical fear was different for patients with no/lower education compared to the reference group, patients with higher/university education [estimate -2.9 (1.3), *p =* .025]. For patients using antidepressants the effect of time (T2 versus T1) on SFQ-s was larger compared to patients not using antidepressants [6.8 (2.1), *p =* .001].

**Table 3:** Predictors of surgical fear.

		SFQ-s		SFQ-l	
Predictor		Estimate (sd)	*p*	Estimate (sd)	*p*
Time	T1	Reference		Reference	
T2		0.18 (.69)	.795	1.51 (.72)	**.036**
T3		.86 (.69)	.214	1.66 (.71)	**.021**
Age		.03 (.09)	.758	-.11 (.08)	.180
Sex		-1.58 (1.50)	.297	-1.59 (1.53)	.300
Education	no/lower	-.97 (2.10)	.645	.15 (2.17)	.946
intermediate		-2.03 (1.65)	.221	-.12 (1.69)	.942
higher/university		Reference		Reference	
Baseline pain		6.01 (2.17)	**.007**	8.64 (2.11)	**< .001**
MOS-SSS (0–100)		-.06 (.03)	.083	NA	
PSS baseline stress (0–40)		.48 (.12)	**< .001**	.47 (.12)	**< .001**
Antidepressants use		3.64 (3.48)	.299	1.24 (3.61)	.733
Presurgical stress T1-T3		NA		-4.34 (2.25)	.056
Interaction with Time					
T2 * no/lower education		-1.15 (1.30)	.379	-1.57 (1.35)	.244
T3 * no/lower education		-2.89 (1.28)	**.025**	-3.27 (1.29)	**.013**
T2 * intermediate education		0.81 (1.00)	.421	-2.15 (1.02)	**.037**
T3 * intermediate education		-.06 (1.01)	.952	-1.66 (1.02)	.104
T2 * antidepressant use		6.83 (2.05)	**.001**	8.04 (2.29)	**.001**
T3 * antidepressant use		1.42 (2.05)	.488	1.54 (2.28)	.499
T2 * presurgical stress T1-T3		NA		.34 (1.37)	.805
T3 * presurgical stress T1-T3		NA		3.55 (1.33)	**.008**
Intercept		6.44 (6.84)	.349	9.86 (5.73)	.089

T1 = one week before surgery, T2 = the day before surgery, T3 = the day of surgery.

Baseline pain = any pain at baseline (NRS ≥ 4). MOS-SSS = Medical Outcomes Study Social Support Survey. PSS baseline stress = Perceived Stress Scale. Presurgical stress T1-T3 = much/very much stress resulting from a major event in the week before surgery.

Significant main effects on SFQ-l were found for baseline pain and perceived stress during the month preceding T1. Similar to the findings with SFQ-s, baseline pain [8.6 (2.1), *p* < .001] and perceived stress during the month before surgery [0.5 (0.1), *p* < .001] were associated with higher SFQ-l scores. Also similar to SFQ-s, we found different effects of time in relation to educational level and use of antidepressants. The effect of time (T3 versus T1) for patients with no/lower educational level was opposite to that of the higher/university level group [estimate -3.3 (1.3), *p =* .013]. The effect of time (T2 versus T1) for patients with intermediate educational level was opposite to that of the higher educational level group [estimate -2.2 (1.0), *p =* .037]. For patients using antidepressants the effect of time (T2 versus T1) on SFQ-l was larger compared to patients not using antidepressants [8.0 (2.3), p 0.001]. Finally, for patients reporting much/very much stress resulting from a major event in the week before surgery, the effect of time (T3 versus T1) on SFQ-l was larger compared to patients with no or little stress [3.6 (1.3), *p =* .008].

### Postoperative evaluation

At T5, we assessed whether any form of preoperative treatment of surgical fear would be appreciated. Most of the patients (72, 77.4%) answered that they had not been afraid of the procedure and did not need any psychological treatment. Thirteen (14%) patients indicated that they had been afraid, but did not need any psychological treatment. Finally, eight (8.6%) patients indicated that they had been afraid and would have appreciated psychological treatment before surgery (five patients had missing data). However, 13 patients in total selected one or more of the proposed preoperative treatment options: more information from the ophthalmologist (8) or anesthesiologist (8), counseling by a psychologist (2), relaxation exercises (2), guided visualization (3), meditation (2), medicinal treatment (3), other (3). Interestingly, a post-hoc analysis revealed that all SFQ-s and SFQ-l scores at T1-3 of the 13 patients asking for preoperative treatment were significantly higher (mean SFQ-s and SFQ-l scores between 12.1 and 14.5) compared with the scores of the patients not asking for support (mean scores between 6.3 and 7.8, all *p* < .05).

## Discussion

The results of this study provide further support for the validity of the SFQ. This is reflected by the high correlations with the fear NRS scores and the moderate but significant correlations with outcome expectancies. As hypothesized, the SFQ was capable of detecting changes in fear over a period from one week before surgery to the day of surgery (SFQ-l only), and in the binocular group also between fear of the first versus the second surgery. However, we found no association with the biological parameters cortisol or alpha-amylase.

Surgical fear levels assessed during the week before elective cataract surgery were low and fear of the short-term aspects of surgery remained stable. However, adequate sensitivity of the SFQ was shown by the detection of small but significant changes in fear over time for SFQ-l, before second eye surgery, and also by the finding that patients indicating any preference for treatment of surgical fear showed significantly higher fear scores. Because changes over time were small, these results suggest that surgical fear scores assessed at different time points in the week before surgery were comparable.

The generally low levels of surgical fear may be due to the refined preoperative procedure of patient assessment and patient information [31]. Also the knowledge that cataract surgery currently is one of the ultimate routine surgical procedures, performed in a modern well-equipped day-surgery setting, might lower preoperative fear levels. Although one previous study on cataract patients reported preoperative STAI anxiety levels above normal compared to norm scores [32], our findings are in line with several other studies on cataract patients [33,34] or fast-track trauma and plastic surgery [35] that also reported low anxiety scores before surgery. The observed low fear scores might also have contributed to the fact that we did not find any association between SFQ and cortisol or alpha-amylase levels. It thus remains the question whether in a population with higher SFQ-scores an association would emerge.

Our data reveal further evidence for the validity of the subscales of the SFQ. Interestingly, for expected recovery, the strongest correlation occurred with the SFQ-l subscale, which includes questions on fear of incomplete and long duration of recovery. The highest correlation with expected pain was found with the SFQ-s, which includes a question on fear of pain, however the difference with SFQ-l was relatively small.

As a secondary aim we examined predictors of the level and preoperative course of surgical fear. In line with previous findings [11], in this population preoperative pain was an overall factor strongly associated with increased SFQ-s and SFQ-l, together with perceived stress in the month before T1. Educational level affected the course of SFQ-s and SFQ-l over time, but the effect sizes were smaller compared to the effect of baseline pain and antidepressant use. Only one other study assessed educational level in relation to the course of surgical fear. This study reported that education had no effect on the course of fear [9]. Previously reported main effects of educational level in relation to fear are conflicting. In one of the samples in our previous report, lower education was associated with lower fear whereas in the other samples lower educated patients had higher fear scores, albeit not significant across all samples [11]. Lower education was also associated with higher fear in another study [36], whereas Ebirim found no differences in surgical fear prevalence between different educational levels [37]. In our study the impact of antidepressant use was substantial. In patients using antidepressants larger effects of time on both SFQ-s and SFQ-l were found. This corresponds with previously described associations between anxiety and depression by other authors [38,39]. An interrelationship between depression and preoperative anxiety assessed two weeks before surgery was found in a study in cardiac surgery patients [40]. However, in contrast to our findings, depression two weeks before surgery was not associated with anxiety assessed one day before surgery.

### Strengths and limitations

The strength of the current study is that further insight in the validity of the SFQ was obtained. The current study further confirms the congruent validity of the SFQ and additionally it reveals that the SFQ is sensitive to detect even small differences in fear, based on time course or other causes. The main limitation of this study is that, as a result of the low fear level in the study population, the ability to assess potential associations between the SFQ and biomarkers cortisol and sAA was restricted.

### Conclusions

Considering the associations with other self-report measures of fear, expected pain and recovery, our data support the validity of the SFQ. Moreover, the SFQ is sensitive to detect small changes in the preoperative course of fear in the week before cataract surgery. However, we found no association between the SFQ and biomarkers cortisol or alpha-amylase. Common factors influencing the course in time of both SFQ-s and SFQ-l are educational level and antidepressant use. Irrespective of time span to surgery, pain and stress at baseline predict increased surgical fear. Except for certain subgroups as mentioned above, the observed variations in fear scores were clinically insignificant, suggesting that in general, surgical fear scores assessed at different time points in the week before surgery are well comparable.

## Supporting information

S1 Fig Boxplot salivary cortisol. Salivary cortisol, nmol/L. ° and * indicate outlier and extreme outlier.
T1 = one week before surgery, T2 = the day before surgery, T3 = the day of surgery,
T5 = the day before the control visit, four weeks after surgery.
M = morning, A = afternoon, E evening, Pre = preoperative, Post = postoperative.
(TIF)

S1 Table Saliva sampling times. T1 = one week before surgery, T2 = the day before surgery, T3 = the day of surgery,
T5 = the day before the control visit, four weeks after surgery.
M = morning, A = afternoon, E evening, Pre = preoperative, Post = postoperative. hh:mm, mean (SD).
(DOC)

S1 File Surgical Fear Questionnaire. (DOC)
