# Supplementary figures and images for: Validity and time course of surgical fear as measured with the Surgical Fear Questionnaire in patients undergoing cataract surgery
Source: PLoS One. 2018 Aug 9;13(8):e0201511. doi: 10.1371/journal.pone.0201511 (PMC6084852; doi:10.1371/journal.pone.0201511)

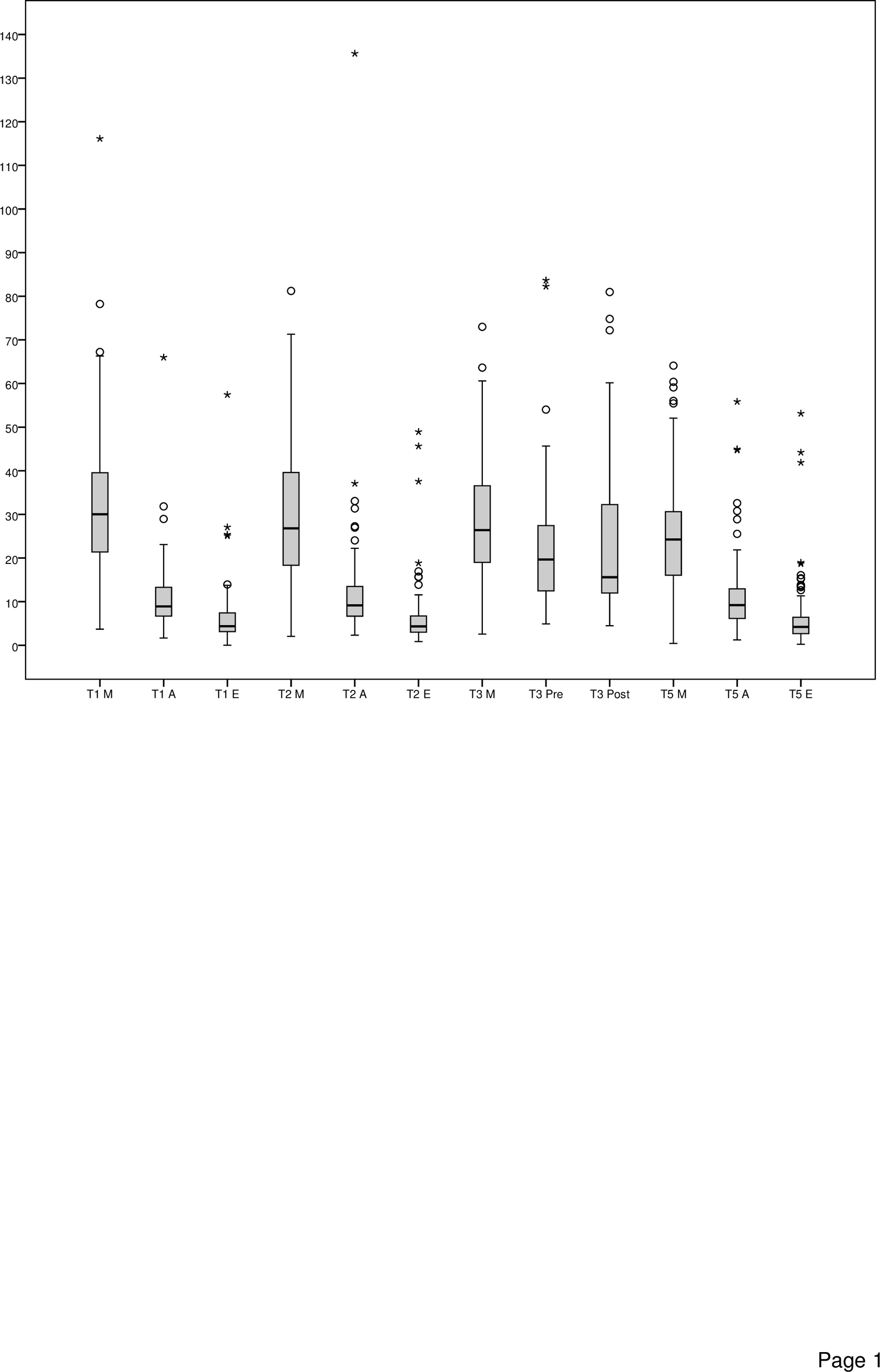

Supplement: S1 Fig — Salivary cortisol, nmol/L. ° and * indicate outlier and extreme outlier. T1 = one week before surgery, T2 = the day before surgery, T3 = the day of surgery, T5 = the day before the control visit, four weeks after surgery. M = morning, A = afternoon, E evening, Pre = preoperative, Post = postoperative. (TIF) [file pone.0201511.s001.tif]
