# Supplementary material for: Validity and time course of surgical fear as measured with the Surgical Fear Questionnaire in patients undergoing cataract surgery
Source: PLoS One. 2018 Aug 9;13(8):e0201511. doi: 10.1371/journal.pone.0201511 (PMC6084852; doi:10.1371/journal.pone.0201511)
Supplement: S1 Table — T1 = one week before surgery, T2 = the day before surgery, T3 = the day of surgery, T5 = the day before the control visit, four weeks after surgery. M = morning, A = afternoon, E evening, Pre = preoperative, Post = postoperative. hh:mm, mean (SD). (DOC) [file pone.0201511.s002.doc]

**Supporting information**

**S1 Table. Saliva sampling times.**

| Sample | T1 M | T1 A | T1 E | T2 M | T2 A | T2 E | T3 M | T3 Pre | T3 Post | T5 M | T5 A | T5 E |
| --- | --- | --- | --- | --- | --- | --- | --- | --- | --- | --- | --- | --- |
| Time | 8:01 | 15:22 | 20:36 | 8:02 | 15:26 | 20:37 | 7:15 | 10:25 | 12:40 | 8:13 | 15:27 | 20:50 |
|  | (1:45) | (1:01) | (0:54) | (0:59) | (0:48) | (0:35) | (0:55) | (1:42) | (2:15) | (1:09) | (1:12) | (1:28) |

T1 = one week before surgery, T2 = the day before surgery, T3 = the day of surgery, T5 = the day before the control visit, four weeks after surgery.

M = morning, A = afternoon, E evening, Pre = preoperative, Post = postoperative.

hh:mm, mean (SD).
